# Further Methodological Aspects in the Prognostic Evaluation of Moxibustion in Stage IV Lung Adenocarcinoma (LUAD-IV)

**DOI:** 10.1177/15347354251378055

**Published:** 2025-09-13

**Authors:** Hegen Li, Veronika Lindberg, Lihua Zhu, Xiange Huang, Jiali Feng, Jan P. A. Baak

To the Editor:

We appreciate the thoughtful comments raised by Wang et al^1^ in their Letter to the Editor regarding time-dependent confounding and other aspects regarding our recent article.^2^

For the sake of clarity, it is important to repeat here the daily clinical background of the use of moxibustion in stage IV non-small cell lung cancer: it is widely used in China and beyond, although adequate proof for its effect is so far lacking, due to methodological shortcomings.^2,3^ Previous studies and meta-analyses had small patient numbers, mixed adeno- and non-adenocarcinomas and even SCLCs, also mixing stage IIIB and IV and limited by short follow-up. Additionally, patients who were deceased within 3 months and therefore never got full western treatments (25%-30% of all NSCLCs), were included, causing major bias and inaccurate results regarding LUAD-IV.

Our prospective observational 2009 to 2018 study prevented these shortcomings. We enrolled from January 1, 2009 to December 2018 412 consecutive LUAD-IV patients with performance score 0 to 1 and 3 to 120 months follow-up, from the Longhua University referral hospital in Shanghai, China. Strong statistical and clinically relevant support was obtained for the survival-improving effect of Moxibustion in the LUAD-IV patients. Multivariate analysis showed it was due to moxibustion and not to follow-up duration. The prognosis-improving effect was dosage-dependent (the more moxa, the better the survival), a strong scientific argument in favor of any intervention.

Methodologically, in contrast to most earlier studies, our propensity score analysis adjusted for bias due to baseline characteristics (age, sex, TNM-IV substages, EGFR mutations, initial TCM syndromes, smoking habits) and sensitivity analyses using multiple balancing methods yielded consistent results. This supported the internal validity of our findings. While no significant association was found between initial TCM syndromes and moxibustion frequency (*P* = .14), the influence of unmeasured time-varying factors cannot be excluded, as we discussed in detail.^2^ We are not aware of published predictors in cancer patients regarding receiving Moxa treatment. However, for other diseases, such as first-time strokes it was found that the choice of TCM treatment was associated with clinical and socioeconomic characteristics and high education.^4^ In addition, Malaysian females with metabolic syndrome were more likely to use TCM.^5^

As described, moxibustion treatments in our patients in 2009 to 2018 were initiated by many different experienced medical oncologists. Moxa therapies were based on evolving TCM syndromes rather than fixed timepoints. This strategy reflects real-world clinical practice, but limited the longitudinal precision required for formal time-dependent modeling. Given these constraints, we consider that our current analytical approach, and the conclusions were correct and appropriate within the observational design.

We agree that examining time-dependent effects may offer valuable insights. Analyzing these in our recently published prospective observational non-interventional study was greatly beyond the possibilities and goals.^2^ Yet, we recommended in the Discussion section^2^ and repeat here that future prospective trials should be specifically designed to capture dynamic clinical changes in relation to treatment exposure and outcomes. We also agree with Wang et al,^1^ that mechanistic studies about moxibustion are important, and cited some of these in our article (Discussion, paragraph 6).^2^

We fully agree and stated that clearly,^2^ that randomized clinical trials are the highest level of proof. We even expressed our hope that others would be able to organize and execute RCTs to prove beyond any doubt that moxibustion in LUAD-IV patients does strongly improve survival. In view of the indications for strong survival improvement with higher dosages, such studies will hopefully also have a full arm with many more moxa treatments than 12. Hopefully, such future RCTs should avoid the serious shortcomings from many earlier moxibustion publications mentioned above in paragraph 2. These strong prognostic heterogeneities may have seriously biased the outcomes and results, also of meta-analyses of stage IV LUAD and other NSCLCs.

Our findings remained consistent across multiple analytic approaches, including propensity score matching, inverse probability weighting, and exact matching. Yet, such methods are inherently limited by the available covariates. Our analysis is methodologically sound within the current data framework, but we also emphasized in the Discussion of our article that definitive evaluation of treatment effects requires randomized controlled trials incorporating comprehensive and up-to-date baseline assessments.


Hegen Li, MD *Longhua University Hospital, Shanghai, China*
Veronika Lindberg, MSc *Lintech AS, Kristiansand, Norway*
Lihua Zhu, MD *Longhua University Hospital, Shanghai, China*
Xiange Huang, MD *Longhua University Hospital, Shanghai, China*
Jiali Feng, MD *Longhua University Hospital, Shanghai, China*
Jan P. A. Baak, MD, PhD, FICP, FRCPath *Stavanger University Hospital, Norway*

